# Increased Adipogenesis of Human Adipose-Derived Stem Cells on Polycaprolactone Fiber Matrices

**DOI:** 10.1371/journal.pone.0113620

**Published:** 2014-11-24

**Authors:** Cecilia Brännmark, Alexandra Paul, Diana Ribeiro, Björn Magnusson, Gabriella Brolén, Annika Enejder, Anna Forslöw

**Affiliations:** 1 Reagent and Assay Development Discovery Sciences R&D, Astra Zeneca, Mölndal, Sweden; 2 Molecular Microscopy Department of Chemical and Biological Engineering, Chalmers University of Technology, Gothenburg, Sweden; Virginia Commonwealth University, United States of America

## Abstract

With accelerating rates of obesity and type 2 diabetes world-wide, interest in studying the adipocyte and adipose tissue is increasing. Human adipose derived stem cells - differentiated to adipocytes *in vitro* - are frequently used as a model system for white adipocytes, as most of their pathways and functions resemble mature adipocytes *in vivo*. However, these cells are not completely like *in vivo* mature adipocytes. Hosting the cells in a more physiologically relevant environment compared to conventional two-dimensional cell culturing on plastic surfaces, can produce spatial cues that drive the cells towards a more mature state. We investigated the adipogenesis of adipose derived stem cells on electro spun polycaprolactone matrices and compared functionality to conventional two-dimensional cultures as well as to human primary mature adipocytes. To assess the degree of adipogenesis we measured cellular glucose-uptake and lipolysis and used a range of different methods to evaluate lipid accumulation. We compared the averaged results from a whole population with the single cell characteristics – studied by coherent anti-Stokes Raman scattering microscopy - to gain a comprehensive picture of the cell phenotypes. In adipose derived stem cells differentiated on a polycaprolactone-fiber matrix; an increased sensitivity in insulin-stimulated glucose uptake was detected when cells were grown on either aligned or random matrices. Furthermore, comparing differentiation of adipose derived stem cells on aligned polycaprolactone-fiber matrixes, to those differentiated in two-dimensional cultures showed, an increase in the cellular lipid accumulation, and hormone sensitive lipase content. In conclusion, we propose an adipocyte cell model created by differentiation of adipose derived stem cells on aligned polycaprolactone-fiber matrices which demonstrates increased maturity, compared to 2D cultured cells.

## Introduction

Human adipose derived stem cells are stem cells from the adipose tissue, that proliferate *in vitro*, and can be stimulated to differentiate into adipocytes [1]–[2], cartilage [3]–[5], or bone [1], [2] cells. When differentiated to adipocytes *in vitro* the cells functionally resemble mature adipocytes in several key aspects, such as lipid accumulation [3]–[5], lipolysis [3]–[5], insulin stimulated glucose uptake [5], [6] and secretion of adipokines [3], [4].

Their similarity to mature adipocytes has led to broad use of differentiated human adipose derived stem cells as an adipocyte model in drug discovery, as well as for physiological investigation of the characteristics and functionality of adipocytes. An important advantage of differentiated adipose derived stem cells compared to mature adipocytes, is that, in contrast to mature adipocytes, they can be cryo-preserved and expanded. However, there are several important differences between differentiated adipose stem cells and freshly isolated human mature adipocytes - for example levels of maximal lipolysis [7] and secretion of TNFα, VEGEF and bFGF [8]. Additionally, the morphology of differentiated adipose derived stem cells, with multiple lipid droplets and a comparatively large cytosolic volume, is different to mature adipocytes that have a large central lipid droplet occupying the vast majority of the cell volume. These differences are indicative of the differentiated adipose derived stem cell being an immature model of the adipocyte.

The development of new protocols and culture conditions for the differentiation of adipose derived stem cells to a more mature state would be beneficial for fundamental studies of adipocyte functions and mechanisms, as well as for *in vitro* drug screening, targeting adipocytes. In the search for improved differentiation protocols for human adipose stem cells many different approaches have been tested and there are now several established methods such as [5], [9]–[11].

To further improve the differentiation of stem cells to adipocytes, efforts have been made to provide the cells with a more physiologically relevant environment by using surface-structure and 3D culture systems [12], the aim being to provide a more *in vivo*-like surrounding in comparison to hard plasticcell culturing plates.Several, recent studies have demonstrated the importance of a cells structural habitat as a driver for phenotypic differentiation [17]–[19]. Various surface-structure and 3D culture systems have been investigated for growing and differentiating preadipocytes/human adipose derived stem cells [13]. These cells have been shown to differentiate to adipocytes in hydro-gels [14]–[20], as floating spheroids [21], on silk scaffolds [22], in hollow bioreactors [23] and on electrospun, synthetic fiber matrices [24]. Such culturing systems have been shown to increased lipid accumulation of adipose derived stem cells. This phenomena can also be observed in other stem cell lineages; for example human bone marrow-derived mesenchymal progenitor cells show increased adipogenesis following culture in 3D systems [25].

For this investigation, a culture system composed of electrospun fibers was selected. An electrospun fiber system is advantagous because it is similar in structure to collagen type I bundles which form the majority of the extra cellular matrix framework in adipose tissue [26] and decellurized human adipose tissue [27]. These types of matrices are porous, which ensures good availability of nutrients and oxygen for cultured cells. Additionally, cells embedded into fibrous structures are also relatively easy to image with optical techniques in comparison to most other 3D-culturing systems. High throughput imaging platforms are common in early drug discovery and therefore it is an advantage if model systems are compatible with such platforms, ie. imaging in microplate format. Furthermore, a commercially available matrix with low batch-to-batch variation is extremely favorable for drug discovery screening.

An attractive material for 3D-culturing is the polymer polycaprolacetone (PCL), the properties of which already meet various medical and physical specifications for safety in clinical applications, and it is biodegradable [28]. PCL has an exceptional ability to form compatible blends, has low toxicity and good biocompatibility [28]. Furthermore, PCL is already a Food and Drug Administration (FDA) approved material for use in the human body as both a drug delivery device and as a suture or adhesion preventing barrier [29], [30]. Use of PCL containing matrices has already been shown to successfully support the differentiation of human adipose derived stem cells [31] into adipocytes and chondorocytes. In addition embryonic stem cells has been reported to differentiate into adipocytes in an electro-spun polymer scaffold [32]. However, electro-spun PCL has not yet been tested for adipogenesis of human adipose derived stem cells.

PCL is suitable for electro-spun matrices and has already been demonstrated to be suitable for culturing of mesenchymal stem cells differentiated into bone [33]. Functionalized PCL fibers with molecular groups that stimulate bone formation, have been reported to improve osteogenesis starting only from mesenchymal stem cells [34], [35] and from adipose stem cells [36], [37].

Taken together, these reports encourage a first study of differentiation capacity and functionality of adipose derived stem cells when promoted towards a linage of adipocytes and cultured on electro-spun PCL matrices. This technique could lead to a viable method of achieving more adipocyte-like phenotypes in stem cell derived adipocyte models, than those currently in use.

To assess adipocyte phenotype *in vitro*, high resolution microscopy techniques are favorable, that can optically investigate the cells whilst they reside in or on a matrix. The optical investigation of cells in a complex matrix is one of the main assets of Coherent Anti-Stokes Raman Scattering (CARS) microscopy, which offers intrinsic chemical selectivity by targeting molecular vibrations [38].

The targeted vibrations are highly characteristic to certain molecules, as they vary with the chemical binding structures presented. This is useful for biological applications as C-H bonds, found in long alkyl chains of triglycerides and fatty acids, can be visualized.

CARS microscopy works by an application of two pulsed lasers onto the sample, overlapping in time and space to drive chosen vibrations. The difference between the two laser frequencies defines the desired oscillation to image the chosen chemical structure. The molecular response can then be read out by a third laser pulse, generating a blue-shifted signal, which is easily separated and usable for laser scanning imaging.

Since the signal is only targeted to a small area of the sample, where the two beams overlap, it is possible to perform 3D sectioning without the use of a pinhole, making this technique suitable for thick samples and cellular structures. The near-infrared light can penetrate further than conventional visible excitation beams used in fluorescence microscopy. Additionally, CARS occurs in the electronic ground state, minimizing potential photodamaging effects on the samples that can occur in more traditional imaging systems [39], [40].

As well as these advantages, the label-free imaging capabilities of CARS microscopy circumvents the difficulty that lipophilic dyes used to stain lipids for visualization also readily binds to the PCL fibers [32]. CARS microscopy has been previously utilized as a non-invasive, label-free technique in lipid biology for the investigation of tumour cells, adipocytes, sebaceous glands and other lipid-rich structures [41].

The aim of this study is to investigate the effect of using aligned and randomly oriented PCL fibers matrices for large cell populations of stem cells differentiated to adipocytes by measuring proliferation, glucose uptake, gene expression and lipolysis. Furthermore, at single cell level, we investigate cell distribution in and on the scaffolds and intracellular lipid stores using CARS microscopy, making comparisons to conventional 2D cell culturing and freshly isolated mature adipocytes.

## Materials and Methods

### Materials

Adipose derived stem cells were cultured and differentiated in tissue culture treated 96-well flat plastic plates (Costar 3595, Corning), PCL fiber plates NanoAligned, thickness ∼10 µM (9602, Nanofiber solutions, Columbus, OH, USA) or NanoECM, thickness ∼20 µM (9601, Nanofiber solutions, Columbus, OH, USA). NanoAligned, is denoted aligned and NanoECM, is denoted random.

### Participants

14 Subject's; 12 females and 2 males with a mean age of 36.8 years (27–49) and a mean BMI of 25.8 (23.6–29.3), undergoing elective surgery, took part in this study. The subjects reported themselves as healthy with no prescribed medications. All subjects gave their written informed consent and all procedures were approved by the ethics board at Gothenburg University and were performed in accordance with the Declaration of Helsinki. N denotes biological repeats and n denotes replicate samples. Adipose-derived stem cells and mature adipocytes were not isolated from the same donors due to technical reasons.

### Adipocyte and adipose derived stem cell isolation

An isolation protocol for extraction of mature adipocytes was adapted from *Danielsson et al.* [42]. Pieces of subcutaneous adipose tissue from the abdomen were extracted under sterile conditions from patients undergoing skin reduction surgery and were then transported in a heated container to the cell isolation lab. The adipose tissue was physically disrupted, and then enzymatically digested with collagenase type II (Worthington, Lakewood, NJ, USA) in 50 ml falcon tubes (Corning New York, NY, USA) in a shaking 37°C incubator (65 rpm). The digested tissue was filtered through a nylon mesh and the mature adipocytes were separated from the other cell types by flotation. The mature adipocytes were rinsed three times in a modified Krebs-Ringer buffer (KRHG), 0.12 M NaCl, 4.7 mM KCl, 2.5 mM CaCl_2_, 1.2 mM MgSO_4_, 1.2 mM KH_2_PO_4_, 20 mM Hepes (pH 7.4), 0, 1% (w/v) fatty acid-free bovine serum albumin, 100 nM phenylisopropyladenosine (Sigma-Aldrich, St. Louis, MO, USA), 0.5 U/mL) adenosine deaminase (Sigma-Aldrich, St. Louis, MO, USA) and 2 mM glucose.

The stromal-vascular fraction was isolated from the filtered, collagenase digested tissue by centrifugation and purified by lysis of the red blood cells using a hypotonic red blood cell lysis buffer (Sigma-Aldrich, St. Louis, MO, USA). The remaining cells were seeded (passage 0), expanded for two passages in BM-1 (ZenBio) supplemented with 10% heat inactivated calf serum (PAA, Houston, TX, USA), penicillin-streptomycin (Invitrogen Carlsbad, CA, USA) and bFGF (Sigma-Aldrich, St. Louis, MO, USA) and stored in −150°C prior to use.

### Differentiation

Human adipose derived stem cells at passage 2 were seeded at 56 250 c/cm^2^ for 2D culture and 112 500 c/cm^2^ for the two categories of matrices in EGM-2NV Bullet kit medium (Lonza, Basel, Switzerland) without gentamycin and cultured at 5% CO_2_ in 37°C. The seeding day was denominated as day 0. On day 1 the medium was changed to differentiation medium BM-1 supplemented with 3% fetal bovine serum (PAA, Houston, TX, USA), 1% Penicillin/Streptomycin, 100 nM insulin (Actrapid, Novo Nordisk, Bagsvaerd, Denmark), 1 µM dexamethason (Sigma-Aldrich, St. Louis, MO, USA), 1 µM pioglitazon (AstraZeneca, London, UK) and 0.5 mM IBMX (Sigma-Aldrich, St. Louis, MO, USA). On day 8 the medium was changed to maturation medium; BM-1 supplemented with fetal bovine serum, 1% penicillin/streptomycin, insulin and dexamethasone (same concentrations as before) and matured until day 14.

### Proliferation

Adipose derived stem cells from three donors were seeded at indicated densities (on either 2D or on matrices), then differentiated using the described protocol, rinsed in phosphate buffer saline (PBS) (Invitrogen, Carlsbad, CA, USA) and fixed at indicated (in the graph) time points using 4% paraformaldehyde (PFA) (HistoLab, Gothenburg, Sweden) for 10 min, stained with Hoechst (350/461 nM) (Invitrogen, Carlsbad, CA, USA). Cells were imaged using a Cellavista system (Synen Tec., Mountain View, CA, USA) and analyzed for nuclear number using the software provided by the manufacturer.

### Live/dead stain

Cells differentiated for a range of time durations, were stained with a live/dead kit containing both green-fluorescent calcein-AM to indicate intracellular esterase activity, and red-fluorescent ethidium homodimer-1 to indicate loss of plasma membrane integrity (Invitrogen, Carlsbad, CA, USA) and imaged using a fluorescence microscope.

### Lipid accumulation

#### Oil-red-o

Differentiated adipose derived stem cells were fixed, using PFA as described above, at indicated time points and stained with oil-red-o (Sigma-Aldrich, St. Louis, MO, USA) then imaged. Oil-o-red was eluted using isopropanol and the absorbance at 490 nm was measured using a Spectramax Pro (Molecular Devices, Sunnyvale, CA, USA).

### Single cell analysis

Cells. Human adipose derived stem cells in passage 2 were seeded in at 112 500cells/cm^2^ on inserts for 24-well plates with aligned or random PCL-fibers or at 56 250 cells/cm^2^ on glass cover slides (170 µm thickness) coated with rat tail collagen type 1 (BD Bioscience, Bedford, MA, USA) and differentiated as described above. The cells were fixed on indicated time points using PFA and stored in PBS until analysis.

#### CARS & MPEF microscopy

Label-free imaging of cells and intracellular lipids was achieved by mapping densities of C-H groups using Coherent Anti-Stokes Raman Scattering (CARS) microscopy. The custom-built setup is described in detail elsewhere [43]. In brief; two picosecond-pulsed laser beams (532 nm and 1064 nm, 7 ps, 76 MHz) were generated by a diode-pumped solid state laser (Nd:Vanadate, 10 W). The 532 nm beam was directed onto an optical parametric oscillator (Levante Emerald OPO, APE Berlin, 690–900 nm), generating a beam at a wavelength of 816.9 nm. The 816.9 and 1064 nm beams, driving the C-H stretching vibration at 2845 cm^−1^, were overlapped in time and space and focussed onto the sample plane of an inverted optical microscope (Eclipse TE2000-E with a C2 Confocal Microscope scanning head, Nikon) using an oil immersion objective (Nikon Plan Fluor, 40×NA 1.30) with a total laser power of 50 mW (at the sample position). The emission of the CARS signal is limited to the high-intensity region of the focal volume, resulting in a spatial resolution of ∼300 nm. Single photon counting detectors from Becker&Hickl GmbH were used to detect three different signals simultaneously. Beside the forward and back emitted CARS signals, intrinsic Multi-Photon Excitation Fluorescence (MPEF) signals were recorded between 495–530 nm. Dichroic mirrors and high optical-density filters were used to separate the signals before the detectors.

CARS/MPEF images were collected with 512×512 pixel image sizes, 5.04 µs pixel dwell time, summed over 5 acquisitions. Multiple planes were sequentially acquired, forming 3D images of the distribution of cells and their lipid stores. The CARS-signals are colour-coded in red and the MPEF-signals shown in green.

Image analysis was done using ImageJ (the National Institutes of Health, USA) and the quantitative voxel analysis used by *Lyn et. al*. [44]. From the thresholded (ImageJ, RenyEnthropie algorithm) CARS images the amount of lipids per cell and the diameter of single lipid droplets were analysed. The MPEF images were used to identify the cells and to measure their sizes.

Lipid droplet diameters were binned in 1 µm bins and the resulting histograms were fitted with a Lorentizian distribution function using OrginPro9 (OriginLab, Northampton, MA, USA).

Cell positions on and in the PCL scaffolds were estimated via their MPFE intensity distribution along the z-axis, while the depth distribution of PCL fibers was determined via their CARS intensity. Both signals were normalized to the maximum. Evaluations were done for a representative z-stack for each scaffold morphology.

3D reconstructions were done with the 3D Viewer tool provided by ImageJ.

### Protein quantification

#### Mature adipocytes

Overnight incubated mature adipocytes were washed in KRHG to remove media, suspended in KRHG at a concentration of 200 µl packed cells/mL and incubated 30 min with 10 nM (-)-N6-(2-phenylisopropyl)adenosine (PIA) (Sigma-Aldrich, St. Louis, MO, USA) and 0.5 U/mL adenosine deaminase (ADA) (Sigma-Aldrich, St. Louis, MO, USA). The cell suspension was subjected to centrifugation through diisonylphtalate and immediately frozen on dry ice until thawed by mixing with Ripa buffer (Sigma-Aldrich, St. Louis, MO, USA), phosphatase inhibitors (phosphostop, Roche) and protease inhibitors (complete mini, Roche) and stored at −20°C until analysis by western blot.

#### Differentiated adipocytes

Fourteenday differentiated adipocytes were washed in PBS and lysed in Ripa buffer and protease and phosphatase inhibitors then kept at −20°C until analyzed using Western blot.

### Western blot

Cell lysate protein concentrations were assessed using a Pierce BCA kit (Thermo Scientific, Waltham, MA, USA) and an equal amount of total protein for each condition was subjected to SDS-PAGE using pre-cast 10% Bis-Tris gels (Invitrogen, Carlsbad, CA, USA), then transferred to a PVDF membrane (Invitrogen, Carlsbad, CA, USA) and immunoblotted with antibodies listed below. Each protein band was normalized to the corresponding anti-actin band which served as a loading control (example blots shown in Figure S1). Samples on different blots could be compared using a standard composed of a mixture of different samples that was run on every gel; samples were adjusted to the intensity of this band. Goat polyclonal anti-actin was from Santa Cruz Biotechnology Inc. (#sc1616) (Dallas, TX, USA) (1∶1000), rabbit polyclonal anti-PKBp473 was from Cell Signaling (#9271) (Danvers, MA, USA) (1∶1000, rabbit polyclonal anti-HSL, was from Cell Signaling (#4107), (Danvers, MA, USA) (1∶1000), rabbit polyclonal anti-HSLp660 was from Cell Signaling (#4126), (Danvers, MA, USA), (1∶1000), rabbit polyclonal anti-insulin receptor was from Santa Cruz Technologies Inc. (#sc-711) (Dallas, TX, USA) (1∶400, rabbit monoclonal anti-perilipin was from Cell Signaling (#9349), (Danvers, MA, USA), (1∶1000). Antibodies were detected using horseradish peroxidase conjugated IgG secondary antibodies rabbit (P0448) or goat (P0449) (1∶1000) (Dako, Glostrup, Denmark) and ECL (Amersham Biosciences, Little Chalfont, Bucks, UK) using chemiluminescence imaging (LAS 3000; Image Gauge v.3.0, Fuji, Tokyo, Japan).

### Statistical analysis

All data are presented as mean ±SEM. Groups were compared using student's t-test with Graph Pad Prism 6 (Graph Pad Software, California, USA). For gene expression specifically, data was analyzed using Analysis of Variance (ANOVA) on the delta Ct values, defining treatment and donor as random and the interaction as random. Comparisons between groups were made using a Student's t-test following the ANOVA. Analyses were conducted using PROC MIXED in SAS 9.2 (SAS, North Carolina, USA). Principal Components Analysis, PCA, was used to give an overview across genes using the R function prcomp (R 3.0.1, r-statistics.com). Data visualization and hierarchical clustering (UPGMA method with Euclidian method for distance measure) was done with TIBCO Spotfire version 5 (Tibco, Maine, USA). Normalization to undifferentiated samples was performed following guidelines from LifeTechnologies [45].

For all analyses, p<0.05 was considered to be significant and indicated with *, p<0.01 was indicated with ** and p<0.001with ***.

### Lipolysis

#### Mature adipocytes

Overnight incubated mature adipocytes were rinsed in KRHG and suspended at 3% cells (v/v) in KRHG. Equal cell volumes were then incubated with the stated concentrations of isoproterenol for 1 hour. Glycerol release in the medium was measured using a glycerol detecting kit (Sigma-Aldrich, St. Louis, MO, USA) according to the manufacturer's protocol.

#### Differentiated adipocytes

Differentiated adipocytes were starved overnight in DMEM (Invitrogen, Carlsbad, CA, USA) supplemented with 1% BSA (Sigma-Aldrich, St. Louis, MO, USA) and 1× penicillin-streptomycin (Sigma-Aldrich, St. Louis, MO, USA), incubated with KRHG for 20 min. Indicated concentrations of isoproterenol (Sigma-Aldrich, St. Louis, MO, USA) were added for 120 min and glycerol release in the medium was measured using a glycerol detection kit (Sigma-Aldrich, St. Louis, MO, USA). The results were related to cell numbers counted with the Cellavista.

### Glucose uptake

#### Mature adipocytes

Mature overnight incubated adipocytes were washed in glucose free buffer, diluted to 200 µl packed cells/mL, incubated 30 min with PIA and ADA. Before the start of the experiment, the adipocytes were incubated with indicated concentrations (in the graph) of insulin or cytocalasin B (Sigma-Aldrich, St. Louis, MO, USA) for 30 min in 37°C.A glucose mixture containing 18 mg/mL glucose and labeled deoxy-D-glucose 2-[1- ^14^C] 100 µCi/ml (PerkinElmer, Waltham, MA, USA) was added to the cells and they were left for a further 60 minute incubation at 37 °C. The incubation was terminated by centrifugation through diisononyl phtalate (Sigma-Aldrich, St. Louis, MO, USA) and immediate freezing using dry ice. The cells were lysed in 100 µl PBS supplemented with 1% SDS and Complete Mini protease inhibitor mix. The lysate was subjected to both scintillation counting and protein concentration determination using a Pierce BCA kit (Thermo Scientific, Waltham, MA, USA).

#### Differentiated adipocytes

Differentiated adipocytes were starved overnight in insulin free media and incubated 3 hours in glucose free DMEM (Invitrogen, Carlsbad, CA, USA) supplemented with 0.4% glucose-containing DMEM (Invitrogen, Carlsbad, CA, USA giving media with final concentrations of 100 µM glucose, 0.1% BSA (Sigma-Aldrich, St. Louis, MO, USA) and 25 mM Hepes (Invitrogen, Carlsbad, CA, USA). Cells were stimulated with indicated concentrations of insulin or cytocalasin B for 30 min. ^14^C-marked D-deoxy-glucose was added and allowed to be taken up for 60 min. Cells were rinsed three times with PBS, then lysed in PBS with 1% SDS and protease inhibitors and subjected to scintillation counting and protein concentration determination using a Pierce BCA kit (Thermo Scientific, Waltham, MA, USA).

### Quantitative real-time RT-PCR

#### Adipose derived stem cells, differentiated adipose derived stem cells and primary mature adipocytes

Adipose derived stem cells from three different donors were plated in a 24 w (aligned/random fiber plate or a 12-well plate (Costar #3512, New York, NY, USA) in BM-1 supplemented with 3% fetal bovine serum and incubated in 37°C overnight. The cells were washed once in PBS and RNA was extracted using a RNA Easy Plus kit (Quiagen, Hilden, Germany). These cells were considered to be undifferentiated adipose derived stem cells.

Fourteen day differentiated adipose derived stem cells, were washed once in PBS and RNA was extracted using a RNA Easy Plus kit.

The RNA concentration was measured using a Nanodrop (Saveen Werner, Limhamn, Sweden) and 0.5 µg of RNA was used for retro transcription with High Capacity cDNA reverse transcription kit (Applied Biosystems, Darmstadt, Germany). 100 ng cDNA per sample was subjected to a custom made TaqMan Low Density Array (Applied Biosystems, Darmstadt, Germany). All samples were run in duplicates.

Expression levels were acquired from the threshold cycle value (Ct) and all statistical calculations are based on deltaCt values (Ct_Gene of interest_ – Ct_Reference genes_), modified from Livak et al [46]. Cycle values for all genes were normalized to the mean of 5 reference genes (LRP10, beta-2-microglobulin, PPIA, GUSB and RPLP0) for each technical replicate. The mean of the reference genes was stable across the various groups.

## Results and Discussion

We aimed to compare the differentiation of human adipose derived stem cells grown as a conventional 2D layer to cells grown on two different electrospun PCL matrices, with either aligned or randomly oriented PCL fibers. We compared the effects of these differentiation environments on human adipose derived stem cells to freshly isolated primary human mature adipocytes. This study is to our knowledge, the first time CARS and MPEF microscopy have been combined with a full set of functional measurements of human adipocytes.

### Cell-distributions differ on the random and aligned matrices

Most methodology used for cell characterization and investigation of functionality, measures average responses of all cells in a well. Here, we complemented population-averaged analysis with single-cell studies using CARS and MPEF microscopy, allowing label-free assessment of the distribution of cells in the scaffolds and of their intracellular lipid stores.

**Figure 1:**
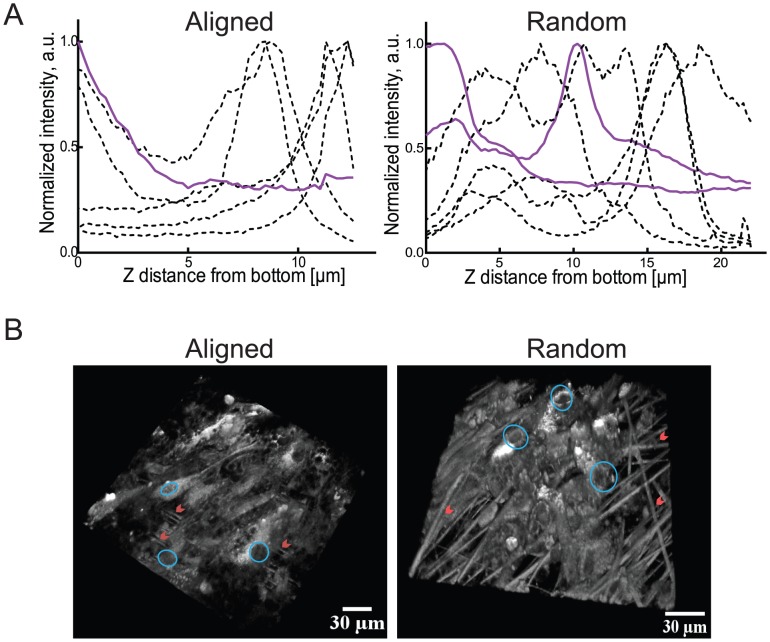
Human adipose derived stem cells growing in PCL fiber matrices. A) Normalized intensities of the CARS (2845 cm^−1^) signal of the fibers (violet, solid) and the MPEF signal (495–530 nm) (black, dashed lines, one line for each cell) of the cells in the matrices in the z dimension for the aligned (left) and random (right) matrices. 5 cells were investigated for each condition, for the random matrix two fibers were shown to visualize the different fiber profiles in this matrix. B) 3D reconstructions of the MPEF signals of the cells on/in the fiber matrices for aligned (left) and random (right) matrices. Nuclei are indicated with circles and fibers with arrowheads (scalebar: 30 µm).

Seeding the stem cells on matrices led to their attachment both inside the matrices and on top of them as demonstrated by CARS/MPEF signal plots which show the normalized intensity versus distance from the well-bottom (Figure 1A). From the example cells measured, it is clear that the cells can either have the bulk of their volume on top of the matrix or they can be localized inside the matrix (Figure 1A and B). Whether the cells sit on or in the matrix, the PCL fiber environments still provided a more physiological environment compared to a 2D culture. CARS analysis also enabled an examination of the PCL fibers themselves in the matrices; the distribution of fibers in the random PCL matrix appeared to be less compact in comparison to the aligned matrix, which could be the reason for the increase of cell localization inside the random matrices.

### Decreased cell proliferation in both random and aligned PCL matrices

Proliferation, in the case of adipose derived stem cells, is in opposition to differentiation, as adipogenesis cannot occur before preadipocytes withdraw from the cell cycle [47], [48]. Inhibition of proliferative events, as well as adipogenesis can also be supported by components of the extracellular matrix [49] and therefore might be influenced by the cell culture environment. This led us to investigate the proliferation of the human adipose derived stem cells in the fiber scaffolds.

**Figure 2:**
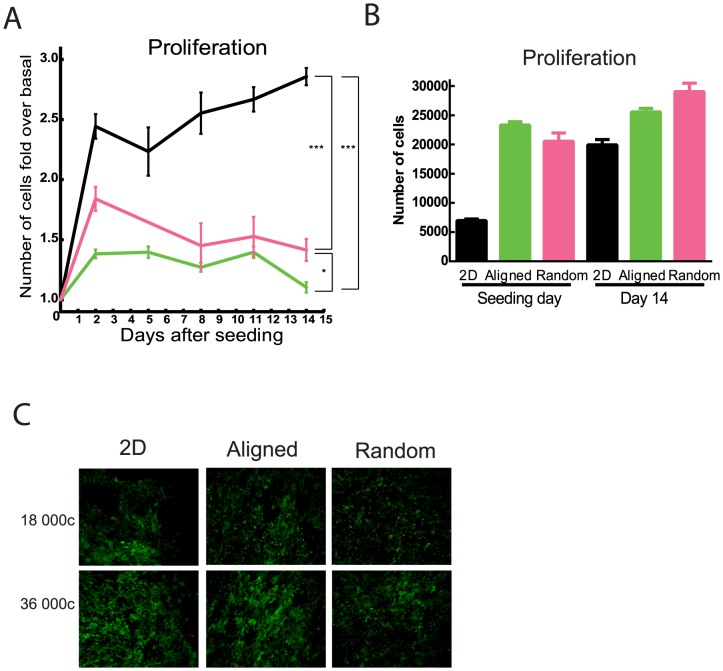
Proliferation of human adipose derived stem cells. P-values are indicated with * when p<0.05 and *** when p<0.001. 2D (black), aligned (green) and random (magenta). A) Proliferation was measured by nuclear count at the indicated time points during the differentiation, in wells seeded with 2D (56250 c/cm^2^), aligned (112500 c/cm^2^) and random (112500 c/cm^2^), n = 10. B) Number of cells was measured as in 1A. C) Live-dead images.14-days differentiated cells were stained live cells (calcein AM) indicated with green and dead cells (Ethidium homodimer) indicated with red thenimaged.

We performed nuclear counts at several time points during differentiation and found significantly lower proliferation in the aligned and random fibers as compared to the 2D plated cells (Figure 2A). A decreased proliferation rate was found over a range of seeding densities (S2A). This result corroborate previous studies reporting that use of PCL nanofiber matrices decreases proliferation of human adipose derived stem cells driven to osteogenesis [35]. Different initial seeding densities were tested in order to ensure that the larger surface area of the fiber-plates compared to conventional, 2D plates, did not influence the results. Seeding densities of 56 250 c/cm^2^ in the control and 112 500 c/cm^2^ in the fiber-plates, resulting in a distribution of 20 000–30 000 cells/well (62 500–93 750 c/cm^2^) after differentiation, were then chosen for all subsequent experiments.

Live-dead staining of the differentiated human adipose derived stem cells indicated no difference in viability between the different culture conditions (Figure 2C), this was consistent over a range of seeding densities and at different time points during the differentiation (S2B).

Preadipocytes need to be withdrawn from the cell cycle before adipose differentiation can start [47] and studies show that differentiation of adipose derived stem cells towards chondrocytes seems to be correlated to a reduction in proliferation [50]. Furthermore, a limitation in space for the cells to proliferate has been suggested to be beneficial for the adipogenesis of preadipocytes [51]. We found a reduction in proliferation of cells grown in both the types of PCL-plates (Figure 2A), which could be due to the matrices giving the cells an environment where they sense spatial restriction. This theory is further supported by the reduced cell area observed in cells in the matrices compared to cells in 2D-plates (S3C). This effect may be because cell adherence to the fibers could resemble cell-to-cell contact, mimicking a more confluent cell layer which would cause the cells to be less prone towards proliferation.

### Increased lipid accumulation in cells on aligned matrices compared to 2D cultures

**Figure 3:**
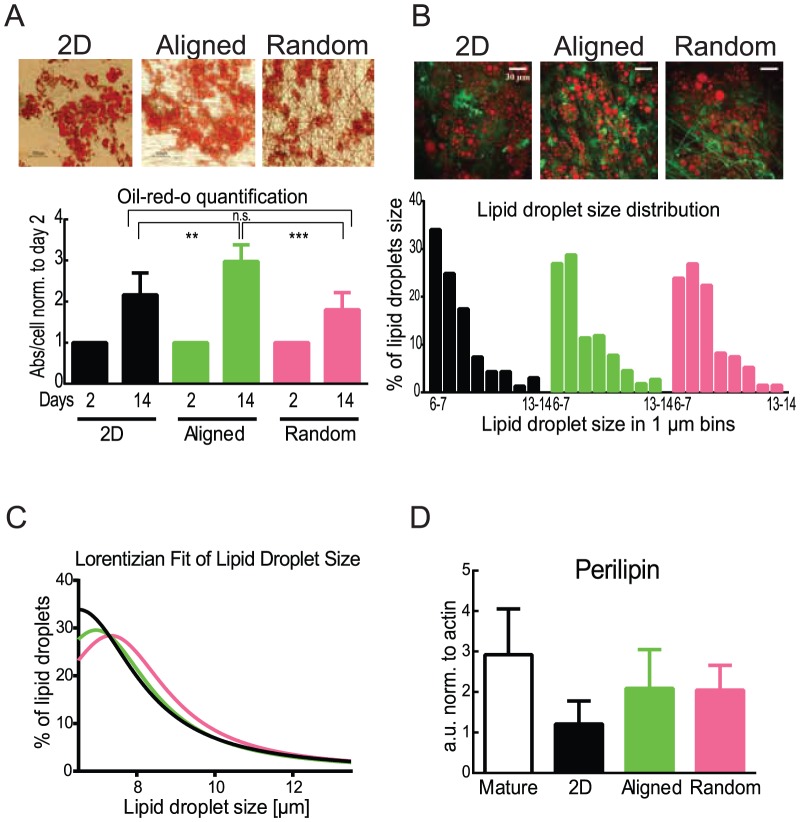
Lipid accumulation during adipogenic differentiation of human preadipocytes in 2D and fiber matrixes. P-values are indicated with ** when p<0.01 and *** when p<0.001, bars without a star were not found to be significantly different from any other bar in the grap. Mature adipocytes (open bars), 2D (black), aligned (green) and random (magenta). A) Upper: 14 days differentiated cells were stained with oil-red-o and imaged in a bright field microscope. Lower: Lipid accumulation was measured as absorbance of eluted oil-o-red at 490 nm at the differentiation day 2 and 14 and normalized to the cell number counted using Cellavista after staining with Hoechst nuclear dye, n = 10. B) Upper: 14 days differentiated cells unstained imaged with CARS (red signals) and MPEF (green signals) microscopy (Order of the images: 2D, aligned, random; scalebar: 30 µm). Lower: Size distribution of lipid droplets in the adipocytes. Presented is the distribution between 6 and 14 µm in 1 µm bins. 30 cells were evaluated per condition. The total droplet number evaluated in each condition was set to 100%. C) Approximation of the droplet size distribution with a Lorentizian function from the data set in 2B. Each data set was normalized to the total number of counted lipid droplets per well in each condition. D) Levels of perilipin in fully differentiated human preadipocytes (N = 3, n = 6) and primary, mature adipocytes (N = 7) measured as a.u. with western blot and normalized to actin content and to a standard as described in the methods section.

Lipid accumulation shown with oil-red-o staining has been used as a golden standard for adipogenesis for years [52], [53]. Accumulation of lipids could be seen in a bright-field microscope after just three to four days of differentiation in all tested culture conditions and after 14 days multiple lipid droplets are clearly visible in all the plate-types (Figure 3A upper).

Oil-red-o pictures suggested more lipid accumulation in cells grown on aligned fibers than in 2D plates and random fibers (Figure 3A upper, S3A and B). We, therefore, quantified the lipid accumulation further from the oil- red-o stained samples, by eluting the staining and measuring the absorbance of the eluate, normalized to the number of nuclei in each well. As the fibers were also stained by the lipophilic oil-red-o, data is normalized to measurements taken on day 2, the time point when we first observed lipid accumulation beginning in the cells. The quantification confirms a significantly higher lipid accumulation in aligned fiber cultures when compared to 2D cultures (Figure 3A lower). Lipid accumulation in cells grown on random fibers, showed no significant difference compared to 2D, but was significantly different to cells grown on aligned fibers (Figure 3A lower). This indicates that the pattern or structure of the fiber matrix, not just the PCL-material, can influence adipogenesis.

Our results demonstrating increased lipid accumulation on cells grown on aligned fibers stands in contrast to findings in embryonic stem cells differentiated to adipocytes that have a similar lipid accumulation in 2D and PCL fiber cultures [32].

High resolution pictures acquired by CARS and MPEF microscopy allow the visualization and quantification of cell size (S3C), and of the size distribution of lipid droplets within individual cells (Figures 3B and C). Example images of all three conditions are shown in Figure 3 B, where red represents the CARS signals generated from the lipids and green the intrinsic multi-photon excitation fluorescence generated inside the cells. Many substances in a cell contribute to the intrinsic green fluorescence, for example NADH, folic acid and riboflavin [54].

The CARS signals can be separated from fluorescence signals by their wavelength, allowing individual analysis of both.

The differentiated adipose derived stem cells – in contrast to the mature adipocytes – accumulate a large number of lipid droplets with a broad distribution of size range. There can be as many as 50 individual lipid droplets per cell after 14 days. The mature adipocytes have one large lipid droplet, filling almost the entire cytosol of the cell, with this as our target, our analysis focussed on the quantification of the bigger droplets in the cells. We found the largest droplets typically ranged from 6 to 20 µm in diameter. Thus, we analysed the abundance of droplets in this size range in 10 cells per condition. For cells in the 2D plates and on the aligned matrix we found on average 10 larger droplets per cell, while in cells grown on the random matrix there were less than 5 droplets per cell in the 6–20 µm size range, details can be found in Table 1. In Figure 3B the distribution of the big droplets is displayed as a histogram. Lipid droplets above 14 µm were excluded, as they were rarely found. The distribution was approximated using a Lorentizian function (Figure 1B, Table 1), which indicates there is a tendency towards bigger droplets in cells grown in the matrices.

**Table 1:** Fitting parameters for the Lorentizian distribution in Figure 3C.

	2D	Aligned	Random
**Amplitude [%]**	33.87	29.58	28.43
**Center [µm]**	6.51	6.95	7.33
**Width [µm]**	1.78	1.70	1.76
**Number of analysed droplets**	229	220	134
**Number of analysed cells**	30	30	30

This shift was observed in both the aligned and random matrices with the increase in droplet size being more pronounced in the latter. With a lower total droplet count in the random fiber matrices, leading to an reduced lipid accumulation, the overall lipid content (Figure 3A lower) can be apprehended. Both matrices increase the number of bigger droplets, but only the aligned fiber matrix expands the lipid amount. Hence, no increased lipid content per cell could be observed for cells in the matrices in the single cell studies. This is concurrent with the discrepancy found when comparing the oil-red-o measurements in this investigation to previous studies [32], and could potentially be explained by the co-staining of the PCL-fibers.

To further investigate lipid droplet differences in our culture systems, we measured perilipin the protein lining lipid droplets. Perilipin is important for lipid droplet formation in human adipocytes and its over-expression can promote formation of droplets [55].We found no significant difference in perilipin content between the three differentiation culturing environments and the mature adipocytes (Figure 3D).

Measurements of lipid accumulation are often used as a method to measure differentiation of adipose derived stem cells to adipocytes. However, lipid accumulation is not the only reporter for maturity of an adipocyte. To more accurately appraise differentiation we aimed to assess the functionality of adipose derived stem cells differentiated in 2D plates and on matrices, comparing them to freshly isolated human mature adipocytes.

### Differentiation on PCL matrices causes stem cell adipocytes to mimic mature adipocytes in insulin-stimulated glucose uptake

**Figure 4:**
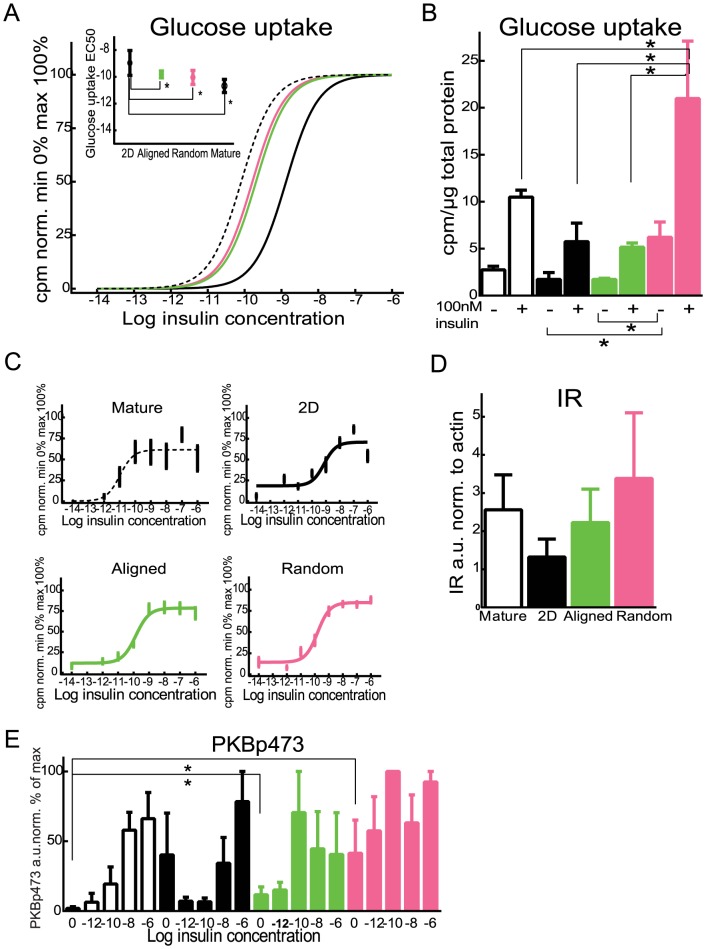
Glucose uptake in differentiated and mature adipocytes. Insulin stimulated glucose transport was measured as uptake of deoxy-D-glucose 2-[1- ^14^C] in response to indicated doses of insulin during 60 min and normalized between 0 and 100% glucose uptake and outliers were excluded using the tukey test in human preadipocytes differentiated in 2D (solid black line, black dots), n = 19 differentiated at three different occations human preadipocytes differentiated on aligned matix (green line and dots), n = 18 differentiated at three different occations, human preadipocytes differentiated on random matrix (magenta line and dots), n = 17 differentiated at three different occations, human mature adipocytes (dashed black line, open circles) measured as a mean of duplicates isolated from 7 different subjects. A) All four data sets normalized to min 0 and max 100% and fitted to the same slope, inserted: EC50 values from the dose-responses, statistical testing was performed using students t-test, * denotes a p-value lower than 0.05. B) Glucose uptake, basally and stimulated with 100 nM insulin, normalized to total protein content. C) All four data sets normalized to min 0 and max 100% and fitted to individual, three parameter slopes. D) Insulin receptor levels measured as arbitrary units and normalized to actin content with western blot. Differentiated human preadipocytes(n = 6, N = 3) or human mature adipocytes N = 7 E) Fully differentiated human preadipocytes N = 3 or human mature adipocytes, N = 6, were stimulated with indicated concentrations of insulin for 10 minutes subjected to SDS-PAGE and western blot, phosphorlylation of serine 473 of PKB measured as arbitrary units and normalized to actin content and maximal phosphorylation was set to 100%.

We first measured the insulin-stimulated glucose uptake of the differentiated adipocytes finding cells grown on random and aligned fibers are significantly more sensitive to insulin than control cells differentiated on 2D (Figure 4A and 4C). The EC50 of the dose-response curves for the PCL matrix differentiated cells was closer to the EC50 of the primary mature adipocytes than to the 2D cells (Figure 4A and C).

The number of transported glucose molecules per cell, measured as cpm/cell was significantly higher in cells grown on random fibers than in the primary mature adipocytes, 2D control or aligned fibers, both basally and after 100 nM insulin stimulus (Figure 4B).

Insulin receptor (IR) levels are known to influence glucose uptake in human adipocytes, for example in type 2 diabetes [56], [57]. However, a comparison of IR protein levels in cells from each of the different conditions, shows no significant differences between the culture conditions (Figure 4D).

We also investigated the phosphorlyation on serine 473 of protein kinase B (PKB) after insulin stimulation as a measure of activity in the insulin signaling pathway. The basal level of phosphorlyated PKB was significantly higher in random and aligned fibers, when compared to mature adipocytes (Figure 4E). No significant difference was found between mature adipocytes and 2D cultured cells at the basal state (Figure 4E). Lower phosphorylation of PKB, in embryonic stem cells differentiated to adipocytes on PCL matrices compared to 2D controls, has previously been reported [32].

In summary, the adipose derived stem cells differentiated on random fibers take up more glucose than mature adipocytes, both basally and after maximal insulin stimulation (Figure 4B). The glucose uptake of mature human adipocytes *in vitro* is increased however, when insulin is absent or very low, when compared to mature human adipocytes *in vivo* [58]. Therefore, a model system with a lower basal glucose uptake and a higher insulin-stimulated glucose uptake than 2D or mature isolated adipocytes would be ideal. These data demonstrate that cells differentiated on the random fiber scaffolds have more functional discrepancies compared to the *in vivo* situation than cells differentiated on aligned fibers, when comparing glucose transport.

### Minimal differences in gene expression between culture systems

To further investigate the impact of the different growth environments on differentiation of the cells, we performed gene expression profiling focusing on 20 genes central to adipocyte function (Table 2, Table S1). Adipose derived stem cells from three donors were subjected to the various culture systems and harvested for quantitative real-time PCR analysis.

**Table 2:** Gene expression quantification.

Symbol	Gene	2D		Random		Aligned	
ACACB	Acetyl-CoA carboxylase, beta	54	(13–231)*	34	(6.5–177)*	42	(10–185)*
ADORA1	Adenosine A1 receptor	0.3	(0.1–1.1)	0.6	(0.3–1.1)	0.7	(0.4–1.1)
ADIPOQ	Adiponectin	7154	(783–65372)**	6088	(576–64343)**	7658	(903–64973)**
PNPLA2	Adipose Triglyceride Lipase (ATGL)	14	(5.2–37)**	12	(4.2–36)*	13	(4.7–36)*
ADRA2A	alpha2-adrenergic receptor	10	(6.5–16)*	11	(6.7–17)*	8.6	(4.3–17)
ADRB1	beta-adrenergic receptor 1	5.3	(0.8–37)	5.1	(0.8–32)	6.5	(1.4–30)
ADRB2	beta-adrenergic receptor 2	3.5	(0.8–16)	5.7	(1.4–23)	5.3	(1–28)
CEBPA	CCAAT/enhancer binding protein, alpha	27	(6.8–104)*	21	(4.3–104)*	23	(5–109)*
CEBPB	CCAAT/enhancer binding protein, beta	2.2	(2–2.5)**	1.9	(1.5–2.5)**	2.1	(1.9–2.4)**
FASN	Fatty acid synthase	7.6	(3–19)*	7.4	(2.5–22)*	7.3	(2.6–20)*
SLC2A1	Glucose Transporter 1 (GLUT1)	3.3	(2.6–4.1)*	3.8	(2.8–5)**	5	(3.3–7.6)**
SLC2A4	Glucose Transporter 4 (GLUT4)	466	(86–2531)*	176	(17 – 1820)*	331	(58–1902)*
INSR	Insulin receptor	2.1	(1.7–2.5)*	1.8	(1.2–2.6)*	1.9	(1.2–3)*
LEP	Leptin	282	(191–418)***	82	(59–112)***##	110	(61–200)***#
LPL	Lipoprotein lipase	2489	(595–10413)**	2330	(490–11082)**	2658	(630–11216)**
MTOR	Mechanistic target of rapamycin	1.8	(1.6–2)*	1.6	(1.4–1.9)	1.7	(1.4–2)
PLIN1	Perilipin	1812	(585–5609)**	947	(200–4490)**	1399	(371–5282)**
PPARGC1A	PGC1-alpha	13	(10–17)***	10	(7.4–14)***	10	(6.9–15)***
PPARGC1B	PGC1-beta	2.1	(1.3–3.4)	2.1	(1–4.4)	2.4	(1.3–4.8)
PPARG	PPAR-gamma	5.7	(3.1–11)*	5.9	(3.8–9.2)*	6.6	(4–11)*

Fold change in gene expression, compared to undifferentiated samples. Gene-expression in human preadipocytes differentited or keept in basal medium (denoted undiff) 14 days on random or aligned fibers or in 2D. Comparisons between groups for individual genes was done using ANOVA followed by pairwise t-tests. Values are mean (+/− SEM) from three donors, run in technical duplicates. * p<0.05, **p<0.01 and ***p<0.001 vs undifferentiated samples. #p<0.05 and ##p<0.01 vs 2D samples. Gene-assay ID's can be found in Table S1.

**Figure 5:**
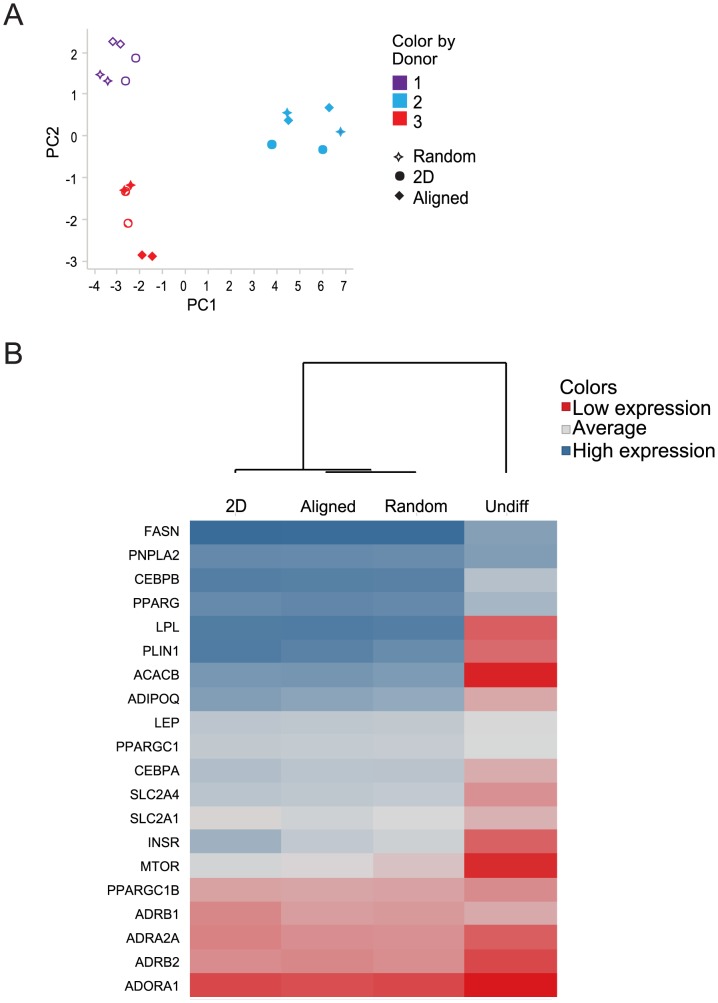
Gene expression. Gene expression in preadipocytes (denoted Undiff) and differentiated human preadipocytes in 2D aligned and random fibers Analyzed data is ΔCt values from technical duplicates. A) PCA analysis of the variation between samples. B) A heatmap was created from mean expression levels in three donors, and the expression profiles were analyzed with hierarchical clustering (UPGMA method with Euclidian method for distance measure).

Initial PCA analysis of the data set from the three groups with differentiated and undifferentiated cells clearly demonstrated that the donor-to-donor variation explained more of the differences in gene expression between samples, compared to the culture systems (Figure 5A). The limited differences in gene expression between culture systems was evident when analyzing genes individually (Table 2). In fact, no gene showed statistically different expression levels between aligned fibers, random fibers and conventional 2D plates, with the exception of leptin, which was 2.6- and 3.4-fold higher in 2D, compared to aligned and random, respectively. The limited differences between culture systems is in contrast to the dramatic changes induced by differentiation itself, which increased the expression level of the majority of analyzed genes. For instance, the classical adipocyte markers Peilipin and Adiponectin were increased ∼1000 and ∼7000-fold, respectively.

This data does show that there are no dramatic differences in expression levels of individual genes used as markers of adipocyte differentiation between the culture systems, especially compared to the normal variation between human subjects. The significant variation between human donors is a strong argument for using multiple donors when studying gene-expression. The fact that we find differences on the functional level and protein levels between cells on the 2D plates and the fiber matrices are unlikely to be due to changes in the expression profiles, but rather to differences in post transcriptional regulation. This clearly argues for the importance of not relying solely on gene-expression analysis for determination of maturation of cells, since small, undetected effects on global transcription might give rise to larger effects on the functionality when numerous proteins in a signaling network are involved. Further exploration of the relationship between gene expression and function in adipocytes is important but beyond the scope of this study.

Despite the absence of dramatic differences on individual gene level, hierarchical clustering can be used to analyze which of the culture systems are most closely related, based on the expression profiles (Figure 5B). Undifferentiated cells showed a very different pattern to all the conditions tested. A comparison between differentiated cells; 2D, random and aligned fiber cultures, shows relatively similar patterns between all conditions. These findings are in line with results from a study on rat adipose derived stem cells, where cells differentiated in a collagen gel had a gene expression profile similar to cells differentiated in 2D [59]. Notably, the random and aligned systems are clustered together, indicating some fiber-mediated analogous effects on the gene expression profile of differentiated cells.

Taken together this data underlines the dramatic effect that differentiation itself has on gene expression, regardless of system, but also indicates that fibers induce some common, but small, transcriptional effects, regardless of orientation.

### Fiber matrices do not induce changes to isoproterenol sensitivity of differentiated adipocytes

**Figure 6:**
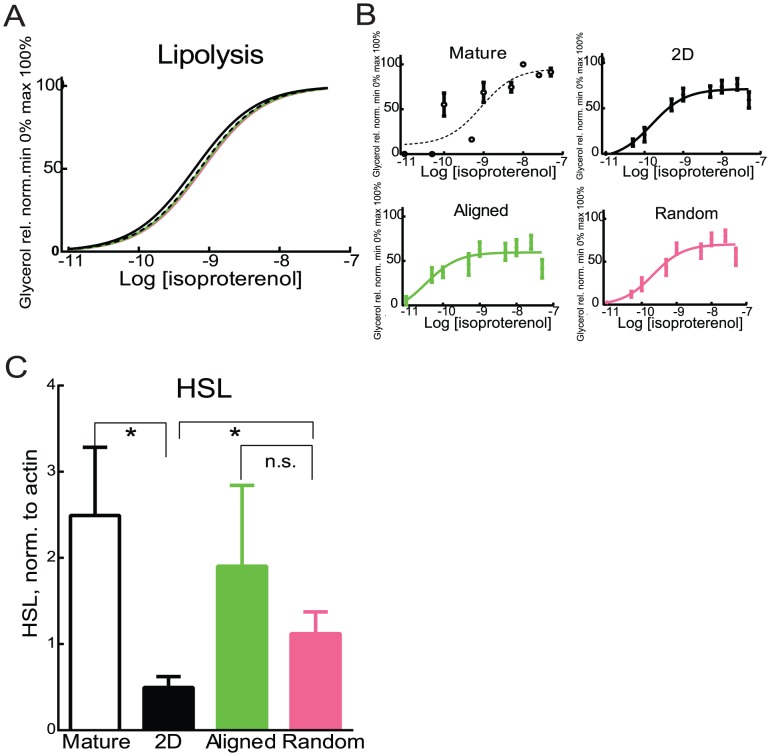
Lipolysis in differentiated and mature adipocytes. Lipolysis was measured as glycerol release and normalized between 0 and 100% to determine EC50 values for human preadipocytes differentiated in 2D (black, solid line), n = 18 differentiated at three separate occasions, human preadipocytes differentiated in aligned (green line), n = 18 differentiated at three separate occasions and human preadipocytes differentiated in random fibers (magenta line), n = 18 differentiated at three separate occasions, mature adipocytes (black dashed line) measured as mean of triplicates isolated from 5 different subjects. A) All four data sets normalized to min 0 and max 100% and fitted to the same slope, no significant differences between the EC50s. B) All four data sets normalized to min 0 and max 100% and fitted to individual, three parameter slopes. C) Fully differentiated human preadipocytes (N = 3, n = 6) or human mature adipocytes (N = 7) were lysed levels of HSL was measured with western blot as arbitrary units normalized to loading control actin. P-value <0.05 is indicated with *, bars or lines without a star were not found to be significantly different from any other bar in the grap.

To further investigate functionality of the differentiated adipocytes, lipolysis measured by glycerol release after isoproterenol stimulation was quantified. The dose response curve for isoproterenol stimulated lipolysis was similar for all differentiated adipocytes and mature adipocytes (Figure 6A–B). This is in line with findings in [32], where embryonic stem cells differentiated to adipocytes on PCL fiber matrices, show no difference in the induction of β-agonist-stimulated (isoproterenol) cAMP production, which would suggest no alteration of lipolysis downstream, unless there are differences in other pathways resulting in a change in lipolysis.

The levels of the rate-limiting enzyme; hormone sensitive lipase (HSL), were significantly higher on random fiber cultured and mature adipocytes in comparison to 2D cultured cells (Figure 6C), whereas the HSL levels in random and aligned cultured cells were not significantly different to mature adipocytes (Figure 6C). Higher HSL content, similar to levels in mature adipocytes, indicate that the adipose derived stem cells differentiated in PCL-fibers are more mature than the cells cultured in 2D plates.

In summary, we find decreased proliferation, increased lipid accumulation, increased HSL levels and more evolved glucose uptake in cells differentiated on PCL fiber matrices compared to conventional cell culture plastic. Potentially, the decreased proliferation can contribute to the increased maturation of the adipose derived stem cells on the fiber plates, as discussed above.

The stiffness of the surface that human adipose derived stem cells adhere to during differentiation has also been coupled to the cells adipogenic potential; a surface mimicking the stiffness of adipose tissue increases adipogenesis in comparison to a harder surface [60]. This might also play a role in the increased lipid accumulation we find in the PCL-fiber systems, as the fibers are significantly softer, 7.1 MPa±1.3 [61], than tissue culture plastic which has a stiffness of about 2–4 GPa [62]. The fiber-plates have a stiffness resembling that of human cartilage [63], whereas human adipose tissue has a stiffness of 17 Pa [62].

There is a difference in how the matrices change the functionality in terms of glucose uptake and lipolysis, affecting one function and not the other. Potentially, lipid handling systems are affected more by environmental cues than the glucose uptake-regulating proteins.

## Conclusions

We investigated the effects on adipogenesis, of human adipose derived stem cells, being differentiated into adipocytes on PCL fiber matrices. We found decreased proliferation, increased lipid accumulation and a closer-to-mature adipocyte EC50 for insulin-stimulated glucose uptake in cells differentiated on aligned PCL fibers compared to 2D plates. Additionally, we found HSL levels and glucose uptake were higher in cells differentiated on random PCL fibers, compared to 2D. In summary these findings indicate enhanced maturation of adipose derived stem cells differentiated on PCL fibers, and we propose use of aligned PCL fiber plates for differentiation of adipose derived stem cells, when a model system is required for glucose uptake of human adipocytes.

## Supporting Information

Figure S1 **Western blots for included antibodies.** Example western blots for the included antibodies as indicated in the Figureure, dilutions can be found in the methods section. Loading control (actin) which was used for normalization is shown for each example blot.
(EPS)

Figure S2 **Proliferation and viability of human adipose derived stem cells in PCL fiber matrices.** A) Cells from donor 1 were seeded 43750 cells/cm^2^ (dotted lines), 56250 cells/cm^2^ (dashed lines) or 112500 cells/cm^2^ (solid lines) in 2D (black lines), aligned (green lines) or random (magenta lines) and differentiated the indicated times. Cells were fixed, stained and counted using the Cellavista. B) Cells seeded in 2D, aligned and random matrices as indicated, at indicated densities and differentiated the indicated times, were stained with green-fluorescent calcein-AM to indicate intracellular esterase activity and red-fluorescent ethidium homodimer-1 to indicate loss of plasma membrane integrity and were imaged with a fluorescence microscope.
(TIF)

Figure S3 **CARS images.** 3D-image of 14-days differentiated human preadipocytes in aligned fibers. B) 3D-image of 14-days differentiated human preadipocytes in random fibers. In both images CARS signals are red-yellow and MPEF signals are blue. C) Cell sizes measured with CARS, n = 30 cells/condition.
(EPS)

Table S1 **Gene-assay ID's.**
(DOCX)

File S1 **Contains cell location data in fiber matices.**
(XLSX)

File S2 ***Contains all cell count, lipid content and cell size data.***
(XLSX)

File S3 **Contain example images for cell count.**
(EPS)

File S4 **Contains all glucose uptake and lipolysis data.**
(XLSX)

File S5 **Contains all western blot data.**
(XLSX)

File S6 **Contains gene expression data.**
(XLSX)

## References

[1] Ogawa R, Mizuno H, Hyakusoku H, Watanabe A, Migita M, et al (2004) Chondrogenic and osteogenic differentiation of adipose-derived stem cells isolated from GFP transgenic mice. J Nippon Med Sch 71:240–241. 15329483 10.1272/jnms.71.240

[2] Halvorsen YD, Franklin D, Bond AL, Hitt DC, Auchter C, et al (2001) Extracellular matrix mineralization and osteoblast gene expression by human adipose tissue-derived stromal cells. Tissue Eng 7:729–741. 11749730 10.1089/107632701753337681

[3] Rodriguez AM, Elabd C, Delteil F, Astier J, Vernochet C, et al (2004) Adipocyte differentiation of multipotent cells established from human adipose tissue. Biochem Biophys Res Commun 315:255–263. 14766202 10.1016/j.bbrc.2004.01.053

[4] Dicker A, Le Blanc K, Astrom G, van Harmelen V, Gotherstrom C, et al (2005) Functional studies of mesenchymal stem cells derived from adult human adipose tissue. Exp Cell Res 308:283–290. 15925364 10.1016/j.yexcr.2005.04.029

[5] Lee MJ, Wu Y, Fried SK (2012) A modified protocol to maximize differentiation of human preadipocytes and improve metabolic phenotypes. Obesity (Silver Spring) 20:2334–2340. 22627913 10.1038/oby.2012.116 PMC4320940

[6] Perrini S, Laviola L, Cignarelli A, Melchiorre M, De Stefano F, et al (2008) Fat depot-related differences in gene expression, adiponectin secretion, and insulin action and signalling in human adipocytes differentiated in vitro from precursor stromal cells. Diabetologia 51:155–164. 17960360 10.1007/s00125-007-0841-7

[7] Langin D, Dicker A, Tavernier G, Hoffstedt J, Mairal A, et al (2005) Adipocyte lipases and defect of lipolysis in human obesity. Diabetes 54:3190–3197. 16249444 10.2337/diabetes.54.11.3190

[8] Blaber SP, Webster RA, Hill CJ, Breen EJ, Kuah D, et al (2012) Analysis of in vitro secretion profiles from adipose-derived cell populations. J Transl Med 10:172. 22913454 10.1186/1479-5876-10-172 PMC3479070

[9] Bunnell BA, Flaat M, Gagliardi C, Patel B, Ripoll C (2008) Adipose-derived stem cells: isolation, expansion and differentiation. Methods 45:115–120. 18593609 10.1016/j.ymeth.2008.03.006 PMC3668445

[10] Yu G, Floyd ZE, Wu X, Hebert T, Halvorsen YD, et al (2011) Adipogenic differentiation of adipose-derived stem cells. Methods Mol Biol 702:193–200. 21082403 10.1007/978-1-61737-960-4_14

[11] Galateanu B, Dinescu S, Cimpean A, Dinischiotu A, Costache M (2012) Modulation of Adipogenic Conditions for Prospective Use of hADSCs in Adipose Tissue Engineering. Int J Mol Sci 13:15881–15900. 23443100 10.3390/ijms131215881 PMC3546668

[12] Guilak F, Cohen DM, Estes BT, Gimble JM, Liedtke W, et al Control of Stem Cell Fate by Physical Interactions with the Extracellular Matrix. Cell Stem Cell 5:17–26. 19570510 10.1016/j.stem.2009.06.016 PMC2768283

[13] Tanzi MC, Fare S (2009) Adipose tissue engineering: state of the art, recent advances and innovative approaches. Expert Rev Med Devices 6:533–551. 19751125 10.1586/erd.09.37

[14] Galateanu B, Dimonie D, Vasile E, Nae S, Cimpean A, et al (2012) Layer-shaped alginate hydrogels enhance the biological performance of human adipose-derived stem cells. BMC Biotechnol 12:35. 22748201 10.1186/1472-6750-12-35 PMC3407005

[15] Kimura Y, Ozeki M, Inamoto T, Tabata Y (2002) Time course of de novo adipogenesis in matrigel by gelatin microspheres incorporating basic fibroblast growth factor. Tissue Eng 8:603–613. 12202000 10.1089/107632702760240526

[16] Uriel S, Huang JJ, Moya ML, Francis ME, Wang R, et al (2008) The role of adipose protein derived hydrogels in adipogenesis. Biomaterials 29:3712–3719. 18571717 10.1016/j.biomaterials.2008.05.028

[17] Halbleib M, Skurk T, de Luca C, von Heimburg D, Hauner H (2003) Tissue engineering of white adipose tissue using hyaluronic acid-based scaffolds. I: in vitro differentiation of human adipocyte precursor cells on scaffolds. Biomaterials 24:3125–3132. 12895585 10.1016/s0142-9612(03)00156-x

[18] Vashi AV, Abberton KM, Thomas GP, Morrison WA, O'Connor AJ, et al (2006) Adipose tissue engineering based on the controlled release of fibroblast growth factor-2 in a collagen matrix. Tissue Eng 12:3035–3043. 17518619 10.1089/ten.2006.12.3035

[19] von Heimburg D, Kuberka M, Rendchen R, Hemmrich K, Rau G, et al (2003) Preadipocyte-loaded collagen scaffolds with enlarged pore size for improved soft tissue engineering. Int J Artif Organs 26:1064–1076. 14738190 10.1177/039139880302601204

[20] Chandler EM, Berglund CM, Lee JS, Polacheck WJ, Gleghorn JP, et al (2011) Stiffness of photocrosslinked RGD-alginate gels regulates adipose progenitor cell behavior. Biotechnol Bioeng 108:1683–1692. 21328324 10.1002/bit.23079

[21] Cheng NC, Wang S, Young TH (2012) The influence of spheroid formation of human adipose-derived stem cells on chitosan films on stemness and differentiation capabilities. Biomaterials 33:1748–1758. 22153870 10.1016/j.biomaterials.2011.11.049

[22] Mauney JR, Nguyen T, Gillen K, Kirker-Head C, Gimble JM, et al (2007) Engineering adipose-like tissue in vitro and in vivo utilizing human bone marrow and adipose-derived mesenchymal stem cells with silk fibroin 3D scaffolds. Biomaterials 28:5280–5290. 17765303 10.1016/j.biomaterials.2007.08.017 PMC2695965

[23] Gerlach JC, Lin YC, Brayfield CA, Minteer DM, Li H, et al (2012) Adipogenesis of human adipose-derived stem cells within three-dimensional hollow fiber-based bioreactors. Tissue Eng Part C Methods 18:54–61. 21902468 10.1089/ten.tec.2011.0216 PMC3245673

[24] Gugerell A, Kober J, Laube T, Walter T, Nürnberger S, et al (2014) Electrospun Poly(ester-Urethane)- and Poly(ester-Urethane-Urea) Fleeces as Promising Tissue Engineering Scaffolds for Adipose-Derived Stem Cells. PLoS ONE 9:e90676. 24594923 10.1371/journal.pone.0090676 PMC3942471

[25] Miyagawa Y, Okita H, Hiroyama M, Sakamoto R, Kobayashi M, et al (2011) A microfabricated scaffold induces the spheroid formation of human bone marrow-derived mesenchymal progenitor cells and promotes efficient adipogenic differentiation. Tissue Eng Part A 17:513–521. 20818998 10.1089/ten.TEA.2009.0810

[26] Chun TH (2012) Peri-adipocyte ECM remodeling in obesity and adipose tissue fibrosis. Adipocyte 1:89–95. 23700517 10.4161/adip.19752 PMC3609086

[27] Wang L, Johnson JA, Zhang Q, Beahm EK (2013) Combining decellularized human adipose tissue extracellular matrix and adipose-derived stem cells for adipose tissue engineering. Acta Biomaterialia 9:8921–8931. 23816649 10.1016/j.actbio.2013.06.035 PMC3965366

[28] Correlo VM, Boesel LF, Pinho E, Costa-Pinto AR, Alves da Silva ML, et al (2009) Melt-based compression-molded scaffolds from chitosan-polyester blends and composites: Morphology and mechanical properties. J Biomed Mater Res A 91:489–504. 18985771 10.1002/jbm.a.32221

[29] Darney PD, Monroe SE, Klaisle CM, Alvarado A (1989) Clinical evaluation of the Capronor contraceptive implant: preliminary report. Am J Obstet Gynecol 160:1292–1295. 2497647 10.1016/s0002-9378(89)80015-8

[30] Allen C, Han J, Yu Y, Maysinger D, Eisenberg A (2000) Polycaprolactone-b-poly(ethylene oxide) copolymer micelles as a delivery vehicle for dihydrotestosterone. J Control Release 63:275–286. 10601723 10.1016/s0168-3659(99)00200-x

[31] Lee JS, Hong JM, Jung JW, Shim JH, Oh JH, et al (2014) 3D printing of composite tissue with complex shape applied to ear regeneration. Biofabrication 6:024103. 24464765 10.1088/1758-5082/6/2/024103

[32] Kang X, Xie Y, Powell HM, James Lee L, Belury MA, et al (2007) Adipogenesis of murine embryonic stem cells in a three-dimensional culture system using electrospun polymer scaffolds. Biomaterials 28:450–458. 16997371 10.1016/j.biomaterials.2006.08.052

[33] Yoshimoto H, Shin YM, Terai H, Vacanti JP (2003) A biodegradable nanofiber scaffold by electrospinning and its potential for bone tissue engineering. Biomaterials 24:2077–2082. 12628828 10.1016/s0142-9612(02)00635-x

[34] Kolambkar YM, Bajin M, Wojtowicz A, Hutmacher DW, Garcia AJ, et al (2014) Nanofiber orientation and surface functionalization modulate human mesenchymal stem cell behavior in vitro. Tissue Eng Part A 20:398–409. 24020454 10.1089/ten.tea.2012.0426 PMC3875145

[35] Pereira IH, Ayres E, Averous L, Schlatter G, Hebraud A, et al (2014) Differentiation of human adipose-derived stem cells seeded on mineralized electrospun co-axial poly(epsilon-caprolactone) (PCL)/gelatin nanofibers. J Mater Sci Mater Med 25:1137–1148. 24378848 10.1007/s10856-013-5133-9

[36] Liao HT, Lee MY, Tsai WW, Wang HC, Lu WC (2013) Osteogenesis of adipose-derived stem cells on polycaprolactone-beta-tricalcium phosphate scaffold fabricated via selective laser sintering and surface coating with collagen type I. J Tissue Eng Regen Med. 10.1002/term.1811 23955935

[37] Yun YP, Kim SJ, Lim YM, Park K, Kim HJ, et al (2014) The effect of alendronate-loaded polycarprolactone nanofibrous scaffolds on osteogenic differentiation of adipose-derived stem cells in bone tissue regeneration. J Biomed Nanotechnol 10:1080–1090. 24749402 10.1166/jbn.2014.1819

[38] Zumbusch A, Holtom GR, Xie XS (1999) Three-Dimensional Vibrational Imaging by Coherent Anti-Stokes Raman Scattering. Physical Review Letters 82:4142–4145.

[39] Evans CL, Xie XS (2008) Coherent anti-stokes Raman scattering microscopy: chemical imaging for biology and medicine. Annu Rev Anal Chem (Palo Alto Calif) 1:883–909. 20636101 10.1146/annurev.anchem.1.031207.112754

[40] Fu Y, Wang H, Shi R, Cheng JX (2006) Characterization of photodamage in coherent anti-Stokes Raman scattering microscopy. Opt Express 14:3942–3951. 19516542 10.1364/oe.14.003942

[41] Le TT, Yue S, Cheng JX (2010) Shedding new light on lipid biology with coherent anti-Stokes Raman scattering microscopy. J Lipid Res 51:3091–3102. 20713649 10.1194/jlr.R008730 PMC2952550

[42] Danielsson A, Ost A, Lystedt E, Kjolhede P, Gustavsson J, et al (2005) Insulin resistance in human adipocytes occurs downstream of IRS1 after surgical cell isolation but at the level of phosphorylation of IRS1 in type 2 diabetes. FEBS J 272:141–151. 15634339 10.1111/j.1432-1033.2004.04396.x

[43] Enejder A, Brackmann C, Svedberg F (2010) Coherent Anti-Stokes Raman Scattering Microscopy of Cellular Lipid Storage. Selected Topics in Quantum Electronics, IEEE Journal of 16:506–515.

[44] Lyn RK, Kennedy DC, Sagan SM, Blais DR, Rouleau Y, et al (2009) Direct imaging of the disruption of hepatitis C virus replication complexes by inhibitors of lipid metabolism. Virology 394:130–142. 19747705 10.1016/j.virol.2009.08.022

[45] LifeTechnologies Guide to Performing Relative Quantitation of Gene Expression Using Real-Time Quantitative PCR.

[46] Livak KJ, Schmittgen TD (2001) Analysis of relative gene expression data using real-time quantitative PCR and the 2(-Delta Delta C(T)) Method. Methods 25:402–408. 11846609 10.1006/meth.2001.1262

[47] Niemelä* SM, Sarkanen JR, Ashammakhi N (2008) Adipose Tissue and Adipocyte Differentiation: Molecular and Cellular Aspects and Tissue Engineering Applications Topics in Tissue Engeneering.

[48] Shi XE, Li YF, Jia L, Ji HL, Song ZY, et al (2014) MicroRNA-199a-5p affects porcine preadipocyte proliferation and differentiation. Int J Mol Sci 15:8526–8538. 24830555 10.3390/ijms15058526 PMC4057746

[49] Myneni VD, Hitomi K, Kaartinen MT (2014) Factor XIII-A transglutaminase promotes plasma fibronectin assembly into preadipocyte extracellular matrix which modulates insulin signalling and preadipocyte proliferation and differentiation. Blood. 10.1182/blood-2013-12-543223 24934257

[50] Im GII, Ko J-Y, Lee JH (2012) Chondrogenesis of Adipose Stem Cells in a Porous Polymer Scaffold: Influence of the Pore Size. Cell Transplantation 21:2397–2405. 22507530 10.3727/096368912X638865

[51] Cristancho AG, Lazar MA (2011) Forming functional fat: a growing understanding of adipocyte differentiation. Nat Rev Mol Cell Biol 12:722–734. 21952300 10.1038/nrm3198 PMC7171550

[52] Pittenger MF, Mackay AM, Beck SC, Jaiswal RK, Douglas R, et al (1999) Multilineage potential of adult human mesenchymal stem cells. Science 284:143–147. 10102814 10.1126/science.284.5411.143

[53] Dominici M, Le Blanc K, Mueller I, Slaper-Cortenbach I, Marini F, et al (2006) Minimal criteria for defining multipotent mesenchymal stromal cells. The International Society for Cellular Therapy position statement. Cytotherapy 8:315–317. 16923606 10.1080/14653240600855905

[54] Zipfel WR, Williams RM, Christie R, Nikitin AY, Hyman BT, et al (2003) Live tissue intrinsic emission microscopy using multiphoton-excited native fluorescence and second harmonic generation. Proc Natl Acad Sci U S A 100:7075–7080. 12756303 10.1073/pnas.0832308100 PMC165832

[55] Grahn TH, Zhang Y, Lee MJ, Sommer AG, Mostoslavsky G, et al (2013) FSP27 and PLIN1 interaction promotes the formation of large lipid droplets in human adipocytes. Biochem Biophys Res Commun 432:296–301. 23399566 10.1016/j.bbrc.2013.01.113 PMC3595328

[56] Brannmark C, Nyman E, Fagerholm S, Bergenholm L, Ekstrand EM, et al (2013) Insulin signaling in type 2 diabetes: experimental and modeling analyses reveal mechanisms of insulin resistance in human adipocytes. J Biol Chem 288:9867–9880. 23400783 10.1074/jbc.M112.432062 PMC3617287

[57] Zhou L, Zhang J, Fang Q, Liu M, Liu X, et al (2009) Autophagy-mediated insulin receptor down-regulation contributes to endoplasmic reticulum stress-induced insulin resistance. Mol Pharmacol 76:596–603. 19541767 10.1124/mol.109.057067 PMC2730390

[58] Nyman E, Brannmark C, Palmer R, Brugard J, Nystrom FH, et al (2011) A hierarchical whole-body modeling approach elucidates the link between in Vitro insulin signaling and in Vivo glucose homeostasis. J Biol Chem 286:26028–26041. 21572040 10.1074/jbc.M110.188987 PMC3138269

[59] Daya S, Loughlin AJ, Macqueen HA (2007) Culture and differentiation of preadipocytes in two-dimensional and three-dimensional in vitro systems. Differentiation 75:360–370. 17286602 10.1111/j.1432-0436.2006.00146.x

[60] Young DA, Choi YS, Engler AJ, Christman KL (2013) Stimulation of adipogenesis of adult adipose-derived stem cells using substrates that mimic the stiffness of adipose tissue. Biomaterials 34:8581–8588. 23953825 10.1016/j.biomaterials.2013.07.103 PMC3786695

[61] Nam J, Johnson J, Lannutti JJ, Agarwal S (2011) Modulation of embryonic mesenchymal progenitor cell differentiation via control over pure mechanical modulus in electrospun nanofibers. Acta Biomater 7:1516–1524. 21109030 10.1016/j.actbio.2010.11.022 PMC3050074

[62] Correia AL, Bissell MJ (2012) The tumor microenvironment is a dominant force in multidrug resistance. Drug Resistance Updates 15:39–49. 22335920 10.1016/j.drup.2012.01.006 PMC3658318

[63] Roy R, Kohles SS, Zaporojan V, Peretti GM, Randolph MA, et al (2004) Analysis of bending behavior of native and engineered auricular and costal cartilage. J Biomed Mater Res A 68:597–602. 14986315 10.1002/jbm.a.10068

