# Supplementary figures and images for: Increased Adipogenesis of Human Adipose-Derived Stem Cells on Polycaprolactone Fiber Matrices
Source: PLoS One. 2014 Nov 24;9(11):e113620. doi: 10.1371/journal.pone.0113620 (PMC4242727; doi:10.1371/journal.pone.0113620)

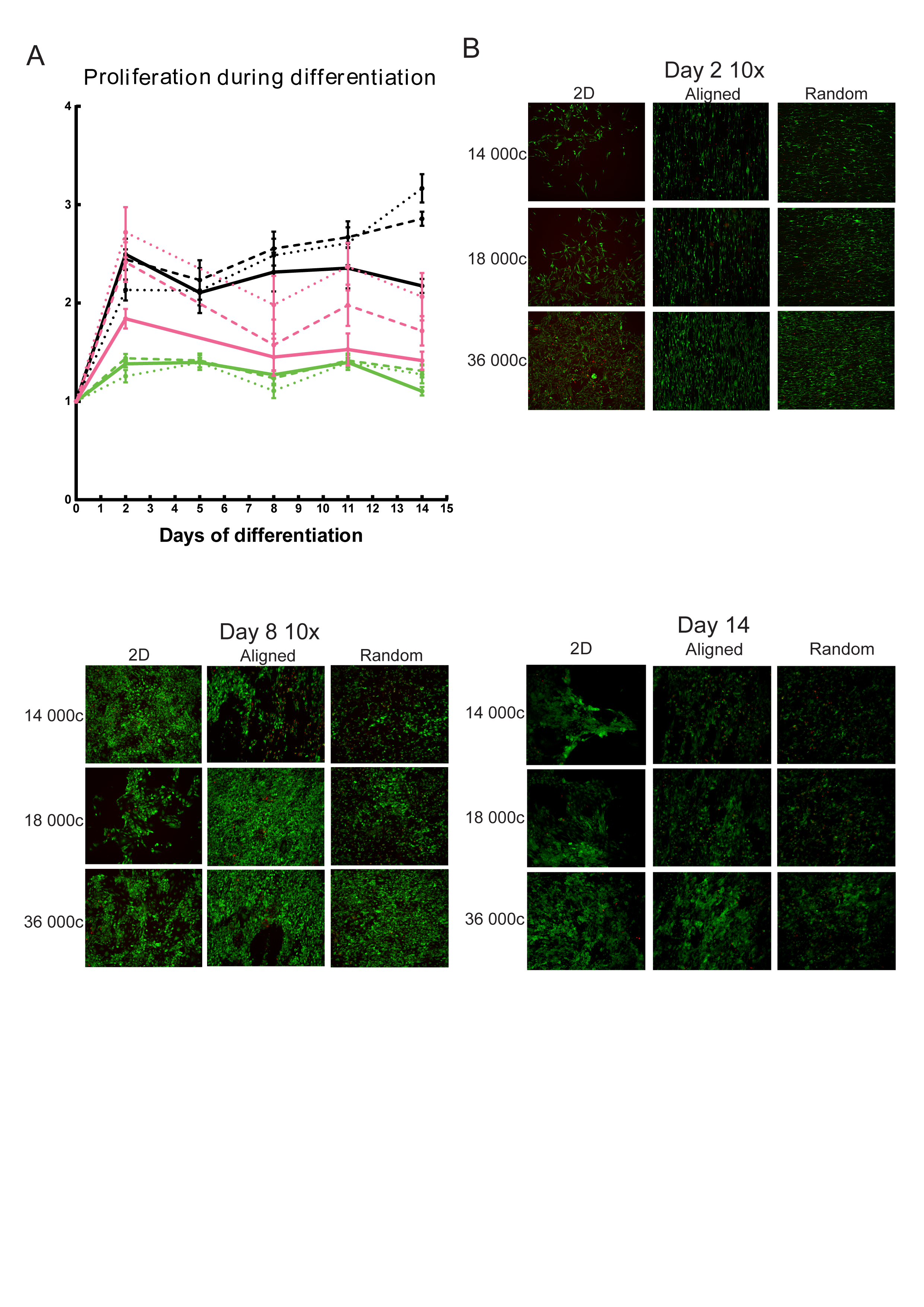

Supplement: Figure S2 — Proliferation and viability of human adipose derived stem cells in PCL fiber matrices. A) Cells from donor 1 were seeded 43750 cells/cm2 (dotted lines), 56250 cells/cm2 (dashed lines) or 112500 cells/cm2 (solid lines) in 2D (black lines), aligned (green lines) or random (magenta lines) and differentiated the indicated times. Cells were fixed, stained and counted using the Cellavista. B) Cells seeded in 2D, aligned and random matrices as indicated, at indicated densities and differentiated the indicated times, were stained with green-fluorescent calcein-AM to indicate intracellular esterase activity and red-fluorescent ethidium homodimer-1 to indicate loss of plasma membrane integrity and were imaged with a fluorescence microscope. (TIF) [file pone.0113620.s002.tif]
