# Supplementary material for: Increased Adipogenesis of Human Adipose-Derived Stem Cells on Polycaprolactone Fiber Matrices
Source: PLoS One. 2014 Nov 24;9(11):e113620. doi: 10.1371/journal.pone.0113620 (PMC4242727; doi:10.1371/journal.pone.0113620)
Supplement: Table S1 — Gene-assay ID's. (DOCX) [file pone.0113620.s004.docx]

| **Symbol** | **Gene** | **Assay-ID** |
| --- | --- | --- |
| ACACB | Acetyl-CoA carboxylase, beta | Hs00153715_m1 |
| ADORA1 | Adenosine A1 receptor | Hs00379752_m1 |
| ADIPOQ | Adiponectin | Hs00605917_m1 |
| PNPLA2 | Adipose Triglyceride Lipase (ATGL) | Hs00386101_m1 |
| ADRA2A | alpha2-adrenergic receptor | Hs00265081_s1 |
| ADRB1 | beta-adrenergic receptor 1 | Hs02330048_s1 |
| ADRB2 | beta-adrenergic receptor 2 | Hs00240532_s1 |
| CEBPA | CCAAT/enhancer binding protein, alpha | Hs00269972_s1 |
| CEBPB | CCAAT/enhancer binding protein, beta | Hs00270923_s1 |
| FASN | Fatty acid synthase | Hs01005622_m1 |
| SLC2A1 | Glucose Transporter 1 (GLUT1) | Hs00892681_m1 |
| SLC2A4 | Glucose Transporter 4 (GLUT4) | Hs00168966_m1 |
| INSR | Insulin receptor | Hs00961554_m1 |
| LEP | Leptin | Hs00174877_m1 |
| LPL | Lipoprotein lipase | Hs00173425_m1 |
| MTOR | Mechanistic target of rapamycin | Hs00234508_m1 |
| PLIN1 | Perilipin | Hs00160173_m1 |
| PPARGC1A | PGC1-alpha | Hs01016719_m1 |
| PPARGC1B | PGC1-beta | Hs00991677_m1 |
| PPARG | PPAR-gamma | Hs01115513_m1 |

Table S4. Gene-assay ID´s .
